# Hypoxia response in *Arabidopsis* roots infected by *Plasmodiophora brassicae* supports the development of clubroot

**DOI:** 10.1186/s12870-016-0941-y

**Published:** 2016-11-11

**Authors:** Antoine Gravot, Gautier Richard, Tanguy Lime, Séverine Lemarié, Mélanie Jubault, Christine Lariagon, Jocelyne Lemoine, Jorge Vicente, Alexandre Robert-Seilaniantz, Michael J. Holdsworth, Maria J. Manzanares-Dauleux

**Affiliations:** 1 IGEPP, AGROCAMPUS OUEST, INRA, Université de Rennes 1, 35650 Le Rheu, France; 2 Division of Plant and Crop Sciences, School of Biosciences, University of Nottingham, Loughborough, LE12 5RD UK

**Keywords:** Ethanol fermentation, Plant gall disease, Clubroot, *Plasmodiophora*, *Arabidopsis*, ADH1, PDC2, N-end rule pathway, Hypoxia, ERFVII

## Abstract

**Background:**

The induction of alcohol fermentation in roots is a plant adaptive response to flooding stress and oxygen deprivation. Available transcriptomic data suggest that fermentation-related genes are also frequently induced in roots infected with gall forming pathogens, but the biological significance of this induction is unclear. In this study, we addressed the role of hypoxia responses in Arabidopsis roots during infection by the clubroot agent *Plasmodiophora brassicae*.

**Results:**

The hypoxia-related gene markers *PYRUVATE DECARBOXYLASE 1* (*PDC1)*, *PYRUVATE DECARBOXYLASE 2* (*PDC2)* and *ALCOHOL DEHYDROGENASE 1* (*ADH1)* were induced during secondary infection by two isolates of *P. brassicae*, eH and e2. *PDC2* was highly induced as soon as 7 days post inoculation (dpi), i.e., before the development of gall symptoms, and GUS staining revealed that *ADH1* induction was localised in infected cortical cells of root galls at 21 dpi. Clubroot symptoms were significantly milder in the *pdc1* and *pdc2* mutants compared with Col-0, but a null T-DNA insertional mutation of *ADH1* did not affect clubroot susceptibility. The Arg/N-end rule pathway of ubiquitin-mediated proteolysis controls oxygen sensing in plants. Mutants of components of this pathway, *ate1 ate2* and *prt6*, that both exhibit constitutive hypoxia responses, showed enhanced clubroot symptoms. In contrast, gall development was reduced in quintuple and sextuple mutants where the activity of all oxygen-sensing Group VII Ethylene Response Factor transcription factors (ERFVIIs) is absent (*erfVII* and *prt6 erfVII*).

**Conclusions:**

Our data demonstrate that the induction of *PDC1* and *PDC2* during the secondary infection of roots by *P. brassicae* contributes positively to clubroot development, and that this is controlled by oxygen-sensing through ERFVIIs. The absence of any major role of *ADH1* in symptom development may also suggest that PDC activity could contribute to the formation of galls through the activation of a PDH bypass.

**Electronic supplementary material:**

The online version of this article (doi:10.1186/s12870-016-0941-y) contains supplementary material, which is available to authorized users.

## Background

Clubroot is a root gall disease of *Brassicaceae* species, caused by the protist *Plasmodiophora brassicae*. The infection process involves a short primary infection of root hairs by zoospores, followed by a secondary phase where plasmodia develop intracellularly in the root cortex for several weeks. During this secondary phase, *P. brassicae* induces hypertrophia and hyperplasia of infected plant cortical cells, leading to the development of galls and to the wilting of the infected plant [1].

Functional genomics approaches have established an increasingly detailed picture of plant signaling and metabolic pathways involved in positive or negative control of clubroot gall development [2]. Untargeted transcriptomic analyses also highlighted additional mechanisms of regulation, but the biological significance of many of these remains uncertain. In this context, Jubault et al. [3] and Schuller et al. [4] pinpointed the induction of ethanol fermentation during secondary infection by *P. brassicae*, and both studies suggested that ethanol fermentation may allow root cells to cope with an oxygen deficit induced by tumor development or by the increased energetic demand in infected cells.

Ethanol fermentation, i.e., conversion of pyruvate into ethanol by the action of pyruvate decarboxylase (PDC) and alcohol dehydrogenase (ADH), is the classical hallmark of root responses to flooding. Under limited oxygen conditions, fermentation allows plant cells to avoid toxic accumulation of pyruvate that would result from the decrease in mitochondrial respiratory activity. This process allows cells to sustain glycolytic fluxes and to meet minimal energetic and metabolic needs to cope with moderate hypoxia constraints. In *Arabidopsis thaliana*, *ADH1*, *PDC1* and *PDC2* have been reported to be important players in flooding-triggered fermentation responses [5–7]. Hypoxia is sensed in plants through the N-end rule pathway of ubiquitin-mediated targeted proteolysis [8, 9]. The five Group VII Ethylene Response Factor transcription factors (RELATED TO APETALA [RAP]2.12, RAP2.2, RAP2.3, HYPOXIA RESPONSIVE ERF [HRE]1 and HRE2) are the only known plant substrates of this pathway, and their oxygen-dependent degradation controls the hypoxia-associated expression of fermentation genes. Oxidation of amino-terminal Cysteine (^OX^Cys) of ERFVII proteins in vivo by oxygen (and nitric oxide) leads to amino-terminal arginylation of ^OX^Cys by ARGINYL TRANSFERASES (ATEs) that allows recognition by an E3 ligase of the N-end rule pathway PROTEOLYSIS (PRT)6 and subsequent ubiquitination and degradation [10–12].

The objectives of the present study were to: 1) specifically document the temporal regulation of ethanol fermentation and other hypoxia-responses during pathogen-induced gall development, and 2) assess the extent to which fermentation, hypoxia-sensing and responses may contribute to the enhancement or reduction of tumorigenic processes. The induction of fermentation during clubroot development was assessed by a combination of RT-qPCR analysis, GUS staining and respiration measurements. The development of clubroot symptoms was evaluated in mutant lines defective for ethanol fermentation, and in mutants of the N-end rule pathway exhibiting constitutive induction (in mutants of the E3 ligase; *prt6* or Arginyl transferase *ate1 ate2*) or constitutive absence of hypoxia responses (*erfVII* and *prt6 erfVII*). The expression of previously described N-end rule and hypoxia regulated genes available in several transcriptome datasets from studies on different tumour-inducing pathogens (*P. brassicae*, the root knot nematode *Meloidogyne javanica* and the crown gall agent *Agrobacterium tumefaciens*) was assessed.

## Results

### Hypoxia-responsive genes are induced early following *P. brassicae* infection, and amplified with club development

**Fig. 1:**
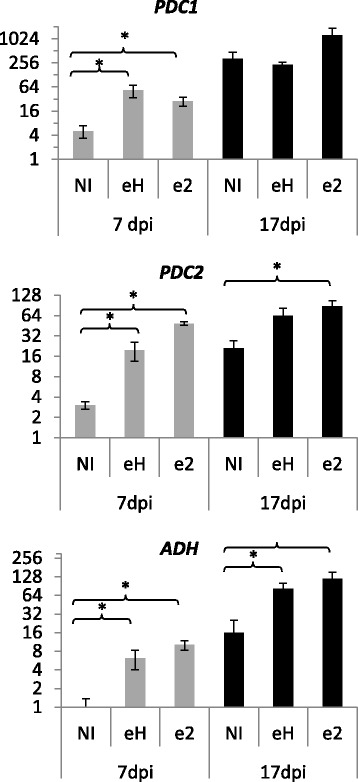
qPCR analysis of the induction of fermentation-related genes during clubroot infection *PDC1* and *PDC2* = *PYRUVATE DECARBOXYLASE 1* and *2. ADH1* = *ALCOHOL DEHYDROGENASE1*. NI = non-inoculated plants. eH & e2 are two isolates of *P. brassicae*. Data are means of 4 independent biological replicates. dpi = days post inoculation. Quantitative data were normalized with the expression of *PP2A* (At1G13320). Y axis is on log scale base 2. Bars represent standard errors. Stars indicate statistically significant differences following Student test (*P* < 0.05)

The *A. thaliana* genotype Col-0 was challenged with two isolates of *P. brassicae*, eH and e2, both virulent on this plant accession [13]. The expression of the fermentation-related genes *PDC1*, *PDC2* and *ADH1* was followed by quantitative RT-qPCR analysis (Fig. 1) at two time-points: an early point at 7 days post-inoculation (dpi), before gall development could be observed, and a later point at 17 dpi, when root galls had clearly developed. The expression of all three genes was significantly increased at 7 dpi in infected plants, compared with low levels of gene expression in a non-inoculated control. At this early stage of secondary infection, the level of induction of *PDC2* was higher in response to inoculation with *P. brassicae* isolate e2 than to inoculation with isolate eH. At 17 dpi, the *PDC2* and *ADH1* expression levels were also induced by infection with both isolates compared with non-inoculated plants.

### GUS staining revealed the induction of *ADH1* in cortical cells of developed root clubs infected by *P. brassicae*

**Fig. 2:**
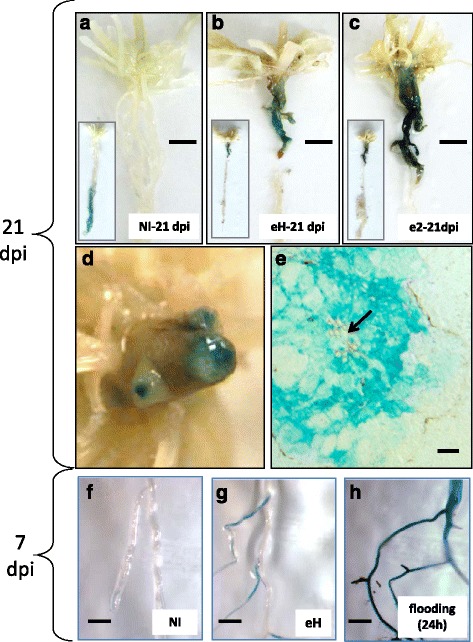
Clubroot-induced regulation of the *ADH1* promoter visualized through GUS staining. **a**-**c** GUS stained upper part of pivotal roots sampled at 21 dpi. **a** non-inoculated plant **b** eH isolate **c** e2 isolate. Rosette leaves were cut after staining for a better visualization of galls. Inserts show the GUS coloration in the lowest part of the root system only in non-inoculated plants. **d** roots infected with eH (21 pi) cut with a razor blade to show the GUS coloration in root cortical tissues **e** microscope observation of GUS coloration in a 3 μm slice of infected root (21 dpi). The arrow indicates the vascular structures. **f**-**h** GUS stained roots sampled at 7 dpi. **f** non-inoculated plants **g** eH isolate, and **h** GUS induction in non-inoculated plants subjected to a 24-h flooding treatment (positive control). Scale bars indicate 4 mm in **a**, **b** and **c**, 100 μm in **e**, and 0.5 mm in **f**-**h**

An *A. thaliana* line in which the promoter of the *ADH1* gene was linked to the GUS reporter gene (*promADH1::GUS*, in a Col-0 genetic background) was used to visualize the expression levels driven by the *ADH1* promoter in infected root tissues, and this line was challenged with eH and e2 isolates (Fig. 2). In non-inoculated plants at 21 dpi (=28 days following germination), *ADH1-promoter* driven expression of GUS was restricted only to the deepest parts of the roots, suggesting that this approach allowed the detection of plant responses to a localized oxygen deficiency in the lowest soil horizon at the time of sampling. At the same time-point in inoculated plants, GUS activity was found to be greatly induced in the clubs. Histological investigation revealed that GUS coloration developed essentially in the inner cell layers subjected to pathogen-triggered hypertrophy and hyperplasia (Fig. 2). Additional investigations were performed at 7 dpi, an early time point of the secondary infection where club development is not yet observable. At this time point, GUS staining patterns were observable on inoculated roots (Fig. 2f-g), mostly on secondary roots, indicating that *ADH1* was induced in infected root tissues before hyperplasia actually started.

### Root respiration activity is not affected during the early secondary phase of clubroot infection

**Fig. 3:**
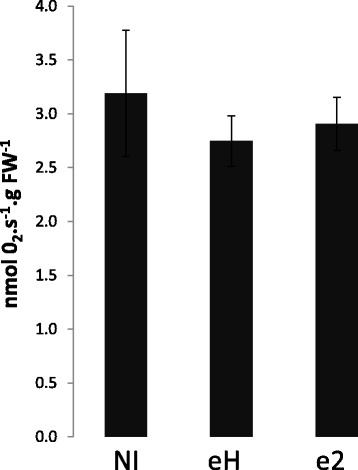
Effect of clubroot infection on the respiration activity in root tissues at 7 dpi. Non-inoculated root samples (NI), and roots from plants inoculated with isolates eH or e2. Data are means of 4 biological repetitions. Bars represent standard errors. No statistical differences were detected between these three experimental conditions

Because the induction of fermentation responses occurred by 7 dpi, before the actual development of galls, we hypothesised that the induction of fermentation-related genes was not likely to be the result of a decline in oxygen diffusion in infect root tissues, which would have led to a reduction in respiration. Nevertheless, this possibility was evaluated by measuring respiration levels in the roots of inoculated and non-inoculated plants at 7 dpi with an oxygraph. The results presented in the Fig. 3 indicate that global respiration in roots was not significantly reduced by infection, suggesting that oxygen diffusion in infected roots was not a major limiting factor at this stage.

### Removal of PCD1 and PDC2 function reduces the development of clubroot

**Fig. 4:**
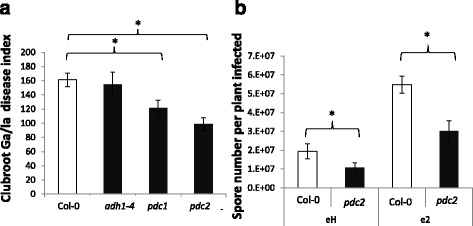
Clubroot susceptibility in lines defective for fermentation-related genes. **a** Impact of mutations *adh1*, *pdc1* and *pdc2* on the development of clubroot symptoms (eH isolate). **b** Impact of mutations on the number of spores of *P. brassicae* per plant at 21 dpi. Data are means of 4 biological repetitions (>12 plant per repetition). Ga/La clubroot disease index is an estimation of the ratio between gall and leaf rosette size from image analysis, as described in Materials and Methods. Bars represent standard errors. Stars represent statistically significant differences between conditions (student *T*-test, *p* < 0.05). The number of large spores (>3 μm) was determined using flow cytometry as described in the material & methods section

Clubroot symptoms in mutants defective for genes involved in the ethanol fermentation pathway are shown in Fig. 4. The mutant lines *pdc1* and *pdc2* displayed reduced clubroot symptoms compared to the wild type Col-0 accession background when inoculated with the isolate eH (Fig. 4a). Disease symptoms were similar in *adh1-4* (from [14]) and Col-0. The mutation *pdc2* also led to a reduction in the number of *P. brassicae* resting spores in infected roots at 21 dpi, with a striking reduction in spore number of the most aggressive isolate, e2 (Fig. 4b). The EMS mutant *adh-R002*, isolated from the Be-0 accession of *A. thaliana* [15], has been widely used to assess the role of fermentation in flooding tolerance. However, we found that the wild accession Be-0 is highly resistant to the isolates eH and e2 (data not shown), and thus the *adh-R002* mutant was not appropriate for the study of *ADH* function in the development of galls.

### Constitutive activation or repression of hypoxia responses in *A. thaliana* mutant lines resulted in enhanced or repressed development of clubs

**Fig. 5:**
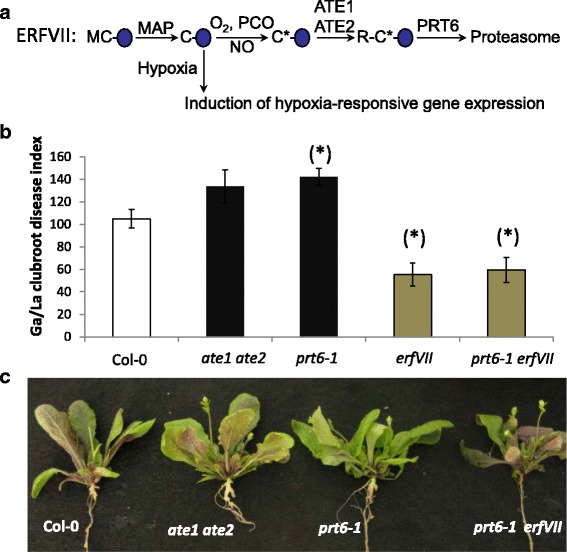
Development of clubroot gall symptoms in Arabidopsis lines with constitutively induced or repressed hypoxia response. **a** Diagrammatical representation of the Arg/N-end rule pathway regulated stability of ERFVII’s and induction of hypoxia-related gene expression. Blue oval, ERFVII substrate proteins showing amino-terminal residues (single letter code); MAP, Met Aminopeptiase; PCO, Plant Cysteine Oxidase; NO, nitric oxide; ATE Arginyl transferase; PRT6, Proteolysis6; C*, oxidised Cysteine. **b** Clubroot symptom index (GA/LA) at 21 dpi. Hypoxia responses are constitutively induced in mutant lines *ate1 ate2* and *prt6-1*, and constitutively repressed in *erfVII* and *prt6-1 erfVII* (*erfVII = rap2.12 rap2.2 rap2.3 hre1 hre2*). Data are means of 3 independent biological repetitions. For each repetition, clubroot symptom index was evaluated from >10 infected plants. Error bars represent SE. Stars indicate statistically significant difference with Col, from the Student test (*P* < 0.05) **c** Illustration of the impact of mutations on clubroot symptoms

Previous work has shown that hypoxia is sensed in plants through the Arg/N-end rule pathway via ERFVII transcription factor substrates. These are destabilised by oxygen in normoxia, through N-end rule activity on amino-terminal oxidised Cys, but stabilised in hypoxia because the N-end rule pathway cannot act on un-oxidised amino-terminal Cys (Fig. 5) [8–11]. In normoxia, *Arabidopsis* mutants of the N-end rule pathway E3 ligase (*prt6*) and arginyl transferase (*ate1 ate2*) that cannot degrade ERFVIIs have constitutive expression of hypoxia and fermentation-related genes [8]. In contrast, genetic removal of ERFVII function reduces fermentation-related gene expression [16]. The *prt6, ate1* ate2 and *erfVII* (*erfVII = rap2.12 rap2.2 rap2.3 hre1 hre2* quintuple mutant) and *prt6 erfVII* mutant lines have been used in recent studies to investigate underexplored physiological functions of hypoxia responses in plant biology [17]. Following challenge with isolate eH of *P. brassicae*, clubroot symptoms were more severe in the constitutive hypoxia response mutants *ate1 ate2* and *prt6*, and milder in *erfVII* and *prt6 erfVII* where the hypoxia response is abrogated (Fig. 5). *erfVII* and *prt6 erfVII* showed the same level of resistance, which indicates the ERFVIIs are the main substrates of the N-end rule pathway involved in this response.

### Hypoxia-transcriptional fingerprints are commonly induced in tumorigenic plant pathogen interactions

**Fig. 6:**
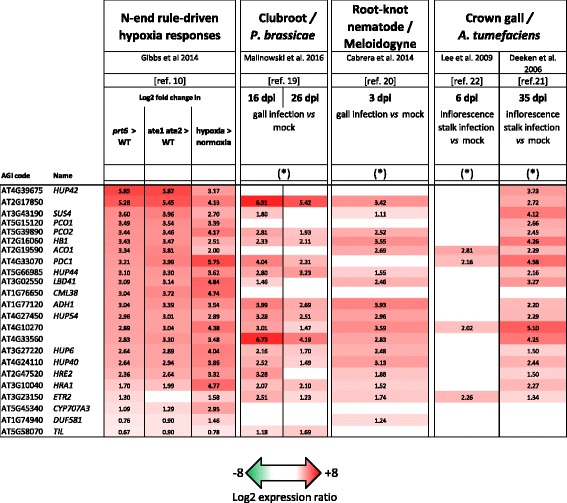
Transcriptional regulation of a set of 23 hypoxia and N-end rule-regulated genes, in *Arabidopsis* plants infected with gall-inducing pathogens. Data are from available transcriptome datasets from the literature [4, 10, 19–22] and are expressed here as log2 ratios between inoculated *vs* non-inoculated conditions for disease responses [4, 19–22], or as indicated for hypoxia and N-end rule driven responses [10]. Stars indicate datasets where only statistically significant regulations are given

A core set of 49 hypoxia-responsive genes has previously been identified in Arabidopsis [18]. Many of these are constitutively up-regulated in N-end rule pathway mutants *prt6* and *ate1 ate2* [8, 10]. We identified 23 genes that are part of this core-hypoxia gene set, and that are also upregulated in *prt6-1* compared to Col-0 WT seedlings (from [10]). The expression of these core-hypoxia and N-end rule regulated genes was investigated in transcriptome datasets available from clubroot studies, and compared with studies with other gall forming pathogens (Fig. 6). We then followed standard procedures (details in Additional files 1, 2, 3 and 4) to process and normalize the publically available RNAseq data from Malinowski et al. [19] (Arrayexpress: E-MTAB-4176), for which Arabidopsis responses to clubroot infection were evaluated in roots and hypocotyls at 16 and 26 dpi (3 biological replicates). A focused analysis (Fig. 6) indicated that a large proportion of N-end rule regulated genes were induced in roots by clubroot infection (17 of 23 genes at 16 dpi, and 14 of 23 genes at 26 dpi). A similar pattern was found in analysed data from clubroot infected hypocotyls (data not shown). Hypoxia responses were also analysed in the transcriptomic responses of hosts to two other gall-forming diseases. A hypoxia transcriptomic fingerprint can be found in the data from root galls induced by root-knot nematodes, at 3 dpi (Fig. 6, data from [20]), with 17 of 23 genes upregulated. In addition, the plant cell response at a late (35 dpi) time-point of *Agrobacterium tumefaciens* infection [21] was associated with the upregulation of 19 of 23 hypoxia gene markers. Among those genes, 5 were among the top 20 with the highest induction levels in *Agrobacterium* crown galls. The dataset of [22] describes an earlier response to *Agrobacterium* at 6 dpi, but even at that early time-point 5 hypoxia responsive genes were significantly induced, including *PDC1*. Altogether, these data suggest that Arg/N-end rule driven hypoxia responses might be a general feature of gall development caused by plant pathogens.

## Discussion

*ADH1* and *PDC1* are commonly used as marker genes for the study of hypoxia responses in plants. The induction of these genes is the emerged face of a (small) iceberg of co-regulated genes that are collectively controlled by the Arg/N-end rule pathway [8, 16]. The data presented in the present work converge to the idea that *P. brassicae* infection significantly induces *ADH1*, *PDC1* and *PDC2* during the secondary infection of roots, and that this response should be viewed as a component of a global stereotypical hypoxia response. Two major factors may induce genuine hypoxia in clubroot infected root tissues: First, the oxygen diffusion rate may be significantly reduced in tumorigenic tissues (previously proposed by [3] and [4]). Second, keeping in mind the observation that *P. brassicae* plasmodia develop intracellularly inside root cortical cells, the plant hypoxia response may result from an intracellular competition for oxygen between the respective mitochondria of *Arabidopsis* and *Plasmodiophora*. Both hypotheses would be consistent with the localisation of *ADH1::GUS* staining in the core infected cells of the root galls (Fig. 2). In our data however, hypoxia-response gene induction was found as soon as 7 dpi, i.e. a time point at the very beginning of root cortical infection where galls are not yet visible. Then, for the earliest time point of the secondary infection, hypoxia responses may result from subtle or localized drops of oxygen availability. Alternatively, one can also not exclude that this response may be triggered by the modulation of other plant-derived factors such as nitric oxide, that also affects Arg/N-end rule degradation of ERFVIIs [10]. If plant hypoxia responses are of benefit to pathogen development, it should be also worthy to envisage that the Arg/N-end rule pathway could be influenced by biochemical effectors of the pathogen, as previous work has shown that this pathway is regulated by small molecules [10]. Additional work would be needed for a clarification of these different possibilities.

Our principal objective in this study was to identify the biological consequences of the induction of fermentative metabolism during clubroot infection. We therefore used appropriate mutant lines to clarify who from the host plant and/or the pathogen would be the payee of ethanol fermentation. The Arabidopsis genome harbours four different PDC encoding genes, but to date only *PDC1* and *PDC2* have been reported to play significant role in hypoxia and flooding responses [5–7]. In the present work, the phenotypes of *pdc1* and *pdc2* mutants indicate that both genes positively contribute to the development of clubroot symptoms. This clearly eliminates the conceivable hypothesis that ethanol biosynthesis by plant cells could act as an antibiotic for inhibiting *P. brassicae* development. Rather, the phenotypes of *pdc* and Arg/N-end rule pathway mutants support a model where hypoxia response benefits disease development and pathogen spore production. This response may be originally a response of the plant to cope with the reduced oxygen availability caused by the infection. As a ‘secondary effect’, the metabolic adaptation to hypoxia may benefit the pathogen, just because any biotrophic pathogen benefits from a host plant that can maintain its metabolic functions as much as possible during the infection process. As discussed above, this conclusion requires confirmation that cell oxygen content actually drops during the development of clubs.

We previously reported [23] that clubroot disease development reaches higher rates in Col-0 when infected plants are cultivated in well-aerated soil substrate. Waterlogging led to the inhibition and restriction of gall development on plant collars, i.e. above the level of water-saturated soil. Thus, from the present study, conducted in well-aerated substrate, it may be inferred that clubroot development is paradoxically at its maximum when hypoxia response is induced in well-aerated infected roots.

ADH activity is a major step of ethanol fermentation because this step allows the regeneration of NAD^+^, thereafter supporting intensive glycolysis flux, and the resulting production of ATP, when respiration is impaired. *ADH1*, being the only ADH encoding gene in Arabidopsis, is actually a key gene to support this mechanism in cells under anoxia [5]. The absence of difference for clubroot symptoms between the *adh1-4* mutant and the wild type was surprising and suggests that, beyond transcriptional regulation, clubroot infection may not activate a genuine anaerobic ethanol fermentation response: 1/ regeneration of NAD^+^ might not be a major stake in root cells infected by *P. brassicae* 2/ PDC-derived acetaldehyde undergoes non-ethanolic fates. Such metabolic features are reminiscent of the ‘PDH bypass’ model, a metabolic pathway also reported as ‘aerobic fermentation’, where acetaldehyde produced by the decarboxylation of pyruvate, is converted to acetyl-CoA, thus furnishing the biosynthesis of fatty acids [24]. The PDH bypass has been experimentally documented in aerobic plant tissues [25, 26], and has been proposed to play a role in the rapid development of actively respiring sporophytic tissues during pollen germination [27]. In the context of clubroot infection, this mechanism would fit with above-described unexpected data: 1/ the fermentation response is triggered at a time point where respiration is apparently unaffected in infected roots 2/ clubroot symptoms are similar in the *adh1-4* mutant and in the wild type. This model would also make sense with the recently reported auxotrophy of *P. brassicae* for fatty acids, suggested by the absence of fatty acid synthase in its genome [28]. Thus, for an efficient clubroot infection, the pathogen may require the activation of metabolic plant features, possibly including a PDH-bypass, which would allow massive synthesis of acetyl-CoA for the synthesis of fatty acids. A careful investigation on carbon fluxes in a series of appropriate mutants would be necessary to test this hypothesis.

## Conclusions

Ethanol fermentation in plant cells has been mostly studied for its role in flooding and hypoxia/anoxia responses. The present work shows that pyruvate decarboxylase genes *PDC1* and *PDC2* support the development of clubroot, and increase the fitness of pathogen through enhancing spore production. The induction of ethanol fermentation genes is part of a prototypical Arg/N-end rule driven hypoxia response, controlled by ERFVII transcription factors, which may play a role in the infection of many gall-forming pathosystems. Further work is needed to assess if hypoxia actually drives the response during the earliest steps of the clubroot infection, and to test the possible role of PDH bypass in regulating clubroot development.

## Methods

### Plant material

All mutant lines were in the Arabidopsis genetic background Columbia. The confirmed homozygous mutant line *pdc1* (SALK_090204C, [29]) harbours a T-DNA in the second exon of the gene *At4g33070*, and was obtained from NASC. The mutant line *pdc2* (SAIL_650_C05) harbours a homozygous insertion in the unique exon of the gene *At5G54960*, and was obtained from NASC (N862662). The mutant *adh1-4* mutant, obtained from NASC (N66116), harbours a knock-out mutation in the gene *At1g77120* generated through Zinc Finger Nuclease as described in [14]. The *promADH1::GUS* line (described in [30]) was kindly provided by Dr. Robert J. Ferl (University of Florida, USA). Arg/N-end rule pathway and *erfVII* mutants were described previously [8, 17].

### Clubroot assays

Clubroot assays were performed as previously described in [31], using isolates eH and e2 of *P. brassicae* described in [13]. Experiments were performed with 3 or 4 independent biological replicates, as specified in the figure legends. Each replicate consisted of at least 12 individual plants and relative spatial disposition of genotypes was randomized in every biological replicate to avoid possible positional effects. All sampled plants were briefly washed with tap water, and then photographed for the evaluation of disease symptoms through image analysis with ImageJ software, as described in [32]. A disease index was calculated as the ratio between the gall area (Ga, in cm^2^) and the square of the longest leaf length (La, in cm^2^) of the rosette, and this ratio was multiplied by a factor of 5000. For each replicate, all of the individual root samples were pooled for further spore quantification of RNA extraction. Spore content in infected roots was evaluated with a flow cytometer as described previously in [23].

### RT- qPCR experiments

The expression of hypoxia-responsive genes *ADH1* (At1g77120), *PDC1* (At4g33070) and *PDC2* (At5G54960) was monitored in the roots of Col-0 under three experimental conditions: 1) non-inoculated, 2) inoculated with isolate eH, and 3) inoculated with isolate e2. Root samples were collected at two time-points (7 and 17 days post-inoculation, dpi), and then immediately frozen in liquid nitrogen prior to storage at −80 °C. RNA extraction and reverse transcription were performed according to [31], using *PP2A3* (At1G13320) as housekeeping reference gene. The primers were as follows: *PP2A*For-TAACGTGGCCAAAATGATGC/ *PP2A*Rev-GTTCTCCACAACCGCTTGGT/ *PDC1.2*For-GGTGGAAGCAACATTGGAGT/ *PDC1.2*Rev-GCTCACTGCTCCCCAATAAG/ *PDC2.2*For-TTGAGGCCATACACAATGGA / *PDC2.2*Rev-GGATTTGGGGGACGACTATT/ *ADH1.2*For-GGTCTTGGTGCTGTTGGTTT/ *ADH1.2*Rev-CTCAGCGATCACCTGTTGAA.

### GUS staining

The *promADH1::GUS* line was challenged with isolate eH and e2 in a bioassay as described above. Plants were sampled at 7 and 21 dpi, and GUS staining (overnight incubation) and histological observations were performed following [32]. To obtain a positive control of *promADH1::GUS expression*, a set of non-inoculated plants was sampled at 6 dpi and maintained in hypoxia conditions for 24 additional hours by dipping inside 50 mL tubes full-filled with tap water, before staining.

### Oxygraph measurements

Respiration was evaluated in samples of roots from plants at 7 dpi using an oxygraph-2 K (Oroboros). The tank of the oxygraph was filled with deionised water and saturated with oxygen by air bubbling. Measurements were calibrated based on room temperature and atmospheric pressure in each experiment. Root samples were immersed in the tank, with a gentle stirring to ensure proper agitation of the medium. The tank was then filled to capacity with additional water to avoid any remaining volume of air above the water. The decrease in oxygen concentration in the water was monitored over a 5 min period. The resulting rate of oxygen consumption was divided by the fresh biomass of the roots.

## Additional files

Additional file 1: Methods used for the analysis of the RNAseq dataset E-MTAB-4176 (from reference [19]). (PDF 44 kb)
Additional file 2: Effect of filtering expressed genes on genes density. Density plot of log(CPM) when all genes are taken into account (Raw data) and after removing genes with a CPM < 1 in at least 6 libraries on the 24 analyzed libraries (Filtered data). The most part of the unexpressed genes is removed after applying this filter, ensuring efficient differentially expressed genes analyzes. (PDF 172 kb)
Additional file 3: Effect of CPM normalization on genes expression profiles. Boxplots representing the expression distribution of the expressed genes (filtered) before and after CPM normalization using TMM method for Normalization Factor calculation. After normalization, the distribution of genes expression of the 24 analyzed samples is similar. (PDF 373 kb)
Additional file 4: Assessment of the biological replicates reproducibility. An unsupervised clustering of sample groups has been performed to verify the likeliness of the biological replicates for each condition. Most groups are correlated and no outlier is detected. All replicates have then been kept for differentially expressed genes analysis. (PDF 8 kb)

## Abbreviations

ADH: Alcohol dehydrogenase
ATE1: Arginine-trna protein transferase 1
ATE2: Arginine-trna protein transferase 2
dpi: Days post inoculation
ERFVIIs: Group VII Ethylene Response Factor transcription factors
PDC1: Pyruvate decarboxylase 1
PDC2: Pyruvate decarboxylase 2
PRT6: Proteolysis 6
RAP: Related to apetala

## References

[1] Kageyama K, Asano T (2009). Life Cycle of *Plasmodiophora brassicae*. J Plant Growth Regul.

[2] Ludwig-Muller J, Prinsen E, Rolfe SA, Scholes JD (2009). Metabolism and Plant Hormone Action During Clubroot Disease. J Plant Growth Regul.

[3] Jubault M, Lariagon C, Taconnat L, Renou J-P, Gravot A, Delourme R (2013). Partial resistance to clubroot in Arabidopsis is based on changes in the host primary metabolism and targeted cell division and expansion capacity. Funct Integr Genomics.

[4] Schuller A, Kehr J, Ludwig-Müller J (2014). Laser microdissection coupled to transcriptional profiling of *Arabidopsis* roots inoculated by *Plasmodiophora brassicae* indicates a role for brassinosteroids in clubroot formation. Plant Cell Physiol.

[5] Ismond KP, Dolferus R, De Pauw M, Dennis ES, Good AG (2003). Enhanced Low Oxygen Survival in *Arabidopsis* through Increased Metabolic Flux in the Fermentative Pathway. Plant Physiol.

[6] Kürsteiner O, Dupuis I, Kuhlemeier C (2003). The *Pyruvate decarboxylase1* Gene of *Arabidopsis* Is Required during Anoxia But Not Other Environmental Stresses. Plant Physiol.

[7] Mithran M, Paparelli E, Novi G, Perata P, Loreti E (2013). Analysis of the role of the pyruvate decarboxylase gene family in *Arabidopsis thaliana* under low-oxygen conditions. Plant Biol.

[8] Gibbs DJ, Lee SC, Isa NM, Gramuglia S, Fukao T, Bassel GW (2011). Homeostatic response to hypoxia is regulated by the N-end rule pathway in plants. Nature.

[9] Licausi F, Kosmacz M, Weits DA, Giuntoli B, Giorgi FM, Voesenek LACJ (2011). Oxygen sensing in plants is mediated by an N-end rule pathway for protein destabilization. Nature.

[10] Gibbs DJ, Isa NM, Movahedi M, Lozano-Juste J, Mendiondo GM, Berckhan S (2014). Nitric Oxide Sensing in Plants Is Mediated by Proteolytic Control of Group VII ERF Transcription Factors. Mol Cell.

[11] Gibbs DJ, Gibbsemail D, Bacardit J, Bachmair A, Holdworth M (2014). The eukaryotic N-end rule pathway: conserved mechanisms and diverse functions. Trends Cell Biol.

[12] Gibbs DJ, Conde JV, Berckhan S, Prasad G, Mendiondo GM, Holdsworth MJ (2015). Group VII Ethylene Response Factors Coordinate Oxygen and Nitric Oxide Signal Transduction and Stress Responses in Plants. Plant Physiol.

[13] Fähling M, Graf H, Siemens J (2003). Pathotype separation of *Plasmodiophora brassicae* by the host plant. J Phytopathol.

[14] Zhang F, Maeder ML, Unger-Wallace E, Hoshaw JP, Reyon D, Christian M (2010). High frequency targeted mutagenesis in *Arabidopsis thaliana* using zinc finger nucleases. Proc Natl Acad Sci U S A.

[15] Dolferus R, Van Den Bossche D, Jacobs M (1990). Sequence analysis of two null-mutant alleles of the single *Arabidopsis Adh* locus. Mol Gen Genet.

[16] Bui LT, Giuntoli B, Kosmacz M, Parlanti S, Licausi F (2015). Constitutively expressed ERF-VII transcription factors redundantly activate the core anaerobic response in *Arabidopsis thaliana*. Plant Sci.

[17] Abbas M, Berckhan S, Rooney DJ, Gibbs DJ, Conde JV, Correia CS (2015). Oxygen Sensing Coordinates Photomorphogenesis to Facilitate Seedling Survival. Curr Biol.

[18] Mustroph A, Zanettia ME, Janga CJH, Holtanb HE, Repettib PP, Galbraithc DW (2009). Profiling translatomes of discrete cell populations resolves altered cellular priorities during hypoxia in Arabidopsis. Proc Natl Acad Sci U S A.

[19] Malinowski R, Novák O, Borhan MH, Spíchal L, Strnad M, Rolfe SA (2016). The role of cytokinins in clubroot disease. Eur J Plant Pathol.

[20] Cabrera J, Bustos R, Favery B, Fenoll C, Escobar C (2014). NEMATIC: a simple and versatile tool for the in silico analysis of plant-nematode interactions. Mol Plant Pathol.

[21] Deeken R, Engelmann JC, Efetova M, Czirjak T, Müller T, Kaiser WM (2006). An integrated view of gene expression and solute profiles of *Arabidopsis* tumors: A genome-wide approach. Plant Cell.

[22] Lee C-W, Efetovaa M, Engelmannb JC, Kramell R, Wasternack C, Ludwig-Müller J (2009). *Agrobacterium tumefaciens* Promotes Tumor Induction by Modulating Pathogen Defense in *Arabidopsis thaliana*. Plant Cell.

[23] Gravot A, Lemarié S, Richard G, Lime T, Lariagon C, Manzanares-Dauleux MJ (2016). Flooding affects the development of *Plasmodiophora brassicae* in *Arabidopsis* roots during the secondary phase of infection. Plant Pathol.

[24] Strommer J (2009). The plant *ADH* gene family. Plant J.

[25] Wei YL, Lin M, Oliver DJ, Schnable PS (2009). The roles of aldehyde dehydrogenases (ALDHs) in the PDH bypass of Arabidopsis. BMC Biochem.

[26] Avidan O, Pick U (2015). Acetyl-CoA synthetase is activated as part of the PDH bypass in the oleaginous green alga Chlorella desiccate. J Exp Bot.

[27] Mellema S, Eichenberger W, Rawyler A, Suter M, Tadege M, Kuhlemeier C (2015). The ethanolic fermentation pathway supports respiration and lipid biosynthesis in tobacco pollen. Plant J.

[28] Schwelm A, Fogelqvist J, Knaust A, Jülke S, Lilja T, Bonilla-Rosso G (2015). The *Plasmodiophora brassicae* genome reveals insights in its life cycle and ancestry of chitin synthases. Sci Rep.

[29] Alonso JM, Stepanova AN, Leisse TJ, Kim CJ, Chen H, Shinn P (2003). Genome-wide insertional mutagenesis of *Arabidopsis thaliana*. Science.

[30] Chung H-J, Ferl RJ (1999). Arabidopsis *Alcohol Dehydrogenase* Expression in Both Shoots and Roots Is Conditioned by Root Growth Environment. Plant Physiol.

[31] Lemarie S, Robert-Seilaniantz A, Lariagon C, Lemoine J, Marnet N, Jubault M (2015). Both the Jasmonic Acid and the Salicylic Acid Pathways Contribute to Resistance to the Biotrophic Clubroot Agent *Plasmodiophora brassicae* in *Arabidopsis*. Plant Cell Physiol.

[32] Gravot A, Deleu C, Wagner G, Lariagon C, Lugan R, Todd C (2012). Arginase induction represses gall development during clubroot infection in *Arabidopsis*. Plant Cell Physiol.

