# Supplementary material for: Hypoxia response in Arabidopsis roots infected by Plasmodiophora brassicae supports the development of clubroot
Source: BMC Plant Biol. 2016 Nov 11;16:251. doi: 10.1186/s12870-016-0941-y (PMC5106811; doi:10.1186/s12870-016-0941-y)
Supplement: Additional file 1: — Methods used for the analysis of the RNAseq dataset E-MTAB-4176 (from reference [19]). (PDF 44 kb) [file 12870_2016_941_MOESM1_ESM.pdf]

Additional file 1: RNA-seq data analysis

Gene expression in *A. thaliana* hypocotyl or roots structures infected or not by clubroot at 16 or 26 days after inoculation with 3 biological replicates was analyzed using an already published dataset accessible at Array Express with the following accession number: E-MTAB-4176 (Malinowski et al. 2016). The paired-end libraries were mapped to the TAIR10 genome annotation and assembly of *A. thaliana* using STAR (Dobin et al., 2013) with the following parameters: --outFilterMultimapNmax 5 --outFilterMismatchNmax 3 --alignIntronMin 10 --alignIntronMax 50000 --alignMatesGapMax 50000. Fragment counts per genes were estimated by Subread featureCounts (Liao et al., 2014) using default parameters. Using R (R Core Team, 2014) with the limma (Phipson et al., 2016; Ritchie et al., 2015) and edgeR (Robinson et al., 2010) packages based on Law et al., 2016, these raw fragment counts were converted to counts per million (CPM). Expressed genes have been filtered by removing genes with a CPM<1 in at least 6 libraries on the 24 analyzed libraries. CPMs have been normalized by using edgeR TMM method for Normalization Factor calculation. Heteroscedasticity from count data has been removed using voom precision weights. Finally, differentially expressed genes analyses have been performed by fitting linear models to compare Infected Tissue vs Control Tissue at a given DPI (16dpi, 26dpi on hypocotyl and root structures). Graphics supporting the validation of the afore-described pipeline can be found in Additional files 2-4.

References

Dobin A, Davis CA, Schlesinger F, Drenkow J, Zaleski C, Jha S, Batut P, Chaisson M, Gingeras TR. STAR: ultrafast universal RNA-seq aligner. *Bioinformatics*. 2013;29:15-21.

Law CW, Alhamdoosh M, Su S, Smyth GK, Ritchie ME. RNA-seq analysis is easy as 1-2-3 with limma, Glimma and edgeR. *F1000Research*. 2016;5:1408.

Liao Y, Smyth GK, Shi W. FeatureCounts: an efficient general purpose program for assigning sequence reads to genomic features. *Bioinforma. Oxf. Engl.* 2014;30:923–930.

Malinowski R, Novák O, Borhan MH, Spíchal L, Strnad M, Rolfe SA. The role of cytokinins in clubroot disease. *Eur J Plant Pathol.* 2016;145:543-557.

Phipson B, Lee S, Majewski IJ, Alexander WS, Smyth GK. Robust hyperparameter estimation protects against hypervariable genes and improves power to detect differential expression. *Ann. Appl. Stat.* 2016;10:946–963.

R Core Team, 2014. R: A Language and Environment for Statistical Computing. R Foundation for Statistical Computing, Vienna, Austria.

Ritchie ME, Phipson B, Wu D, Hu Y, Law CW, Shi W, Smyth GK. Limma powers differential expression analyses for RNA-sequencing and microarray studies. *Nucleic Acids Res.* 2014;43:e47.

Robinson MD, McCarthy DJ, Smyth GK. edgeR: a Bioconductor package for differential expression analysis of digital gene expression data. *Bioinforma. Oxf. Engl.* 2010;26:139–140.
