# Supplementary figures and images for: Hypoxia response in Arabidopsis roots infected by Plasmodiophora brassicae supports the development of clubroot
Source: BMC Plant Biol. 2016 Nov 11;16:251. doi: 10.1186/s12870-016-0941-y (PMC5106811; doi:10.1186/s12870-016-0941-y)

Raw data

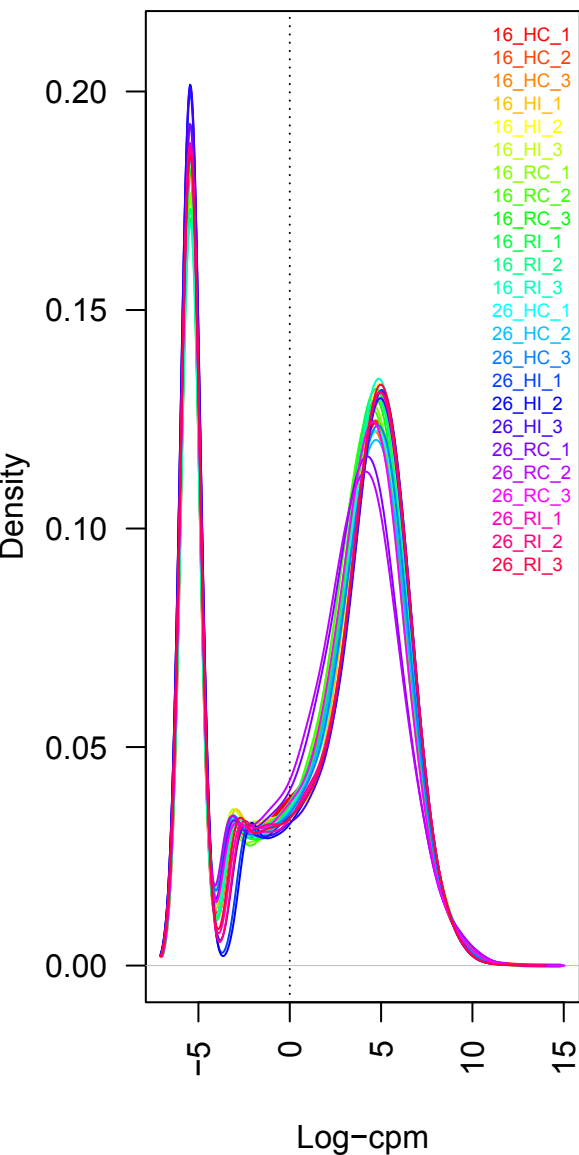

Filtered data

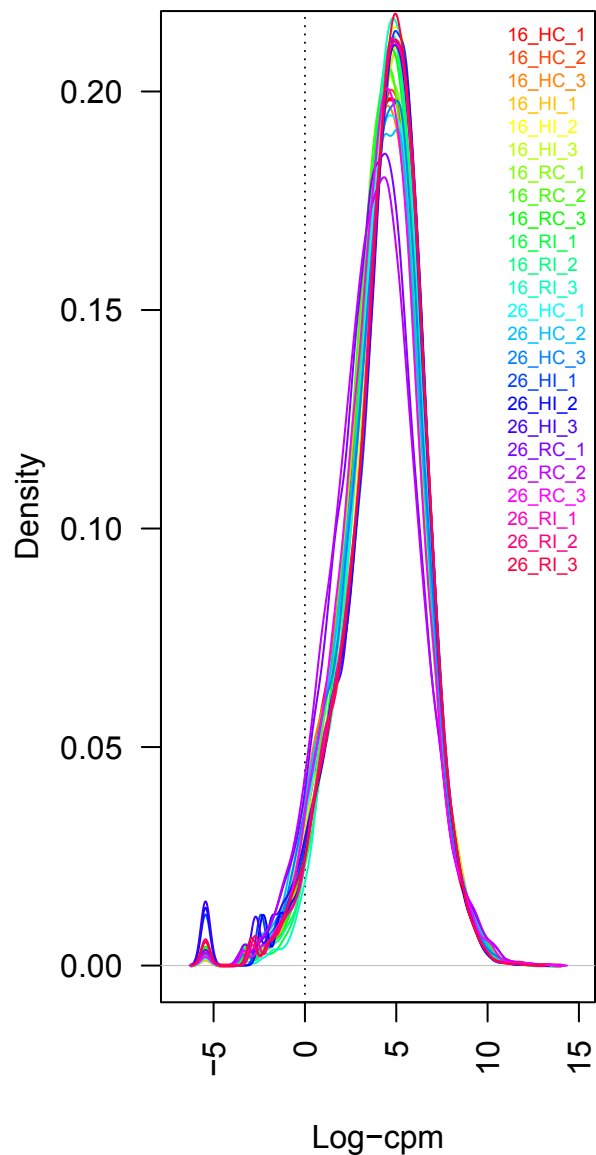

Supplement: Additional file 2: — Effect of filtering expressed genes on genes density. Density plot of log(CPM) when all genes are taken into account (Raw data) and after removing genes with a CPM < 1 in at least 6 libraries on the 24 analyzed libraries (Filtered data). The most part of the unexpressed genes is removed after applying this filter, ensuring efficient differentially expressed genes analyzes. (PDF 172 kb) [file 12870_2016_941_MOESM2_ESM.pdf]

Unnormalised data

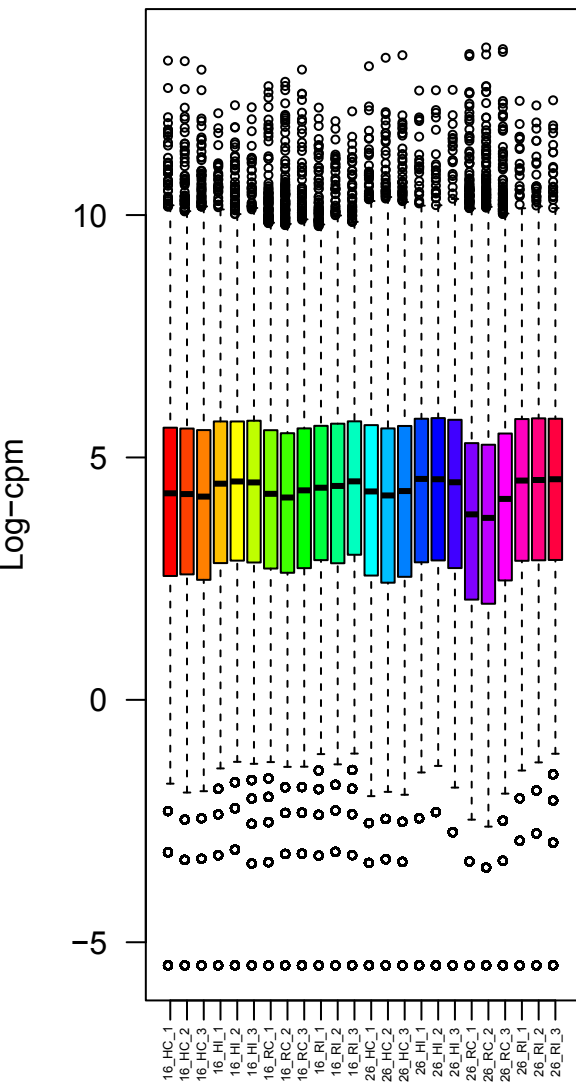

Normalised data

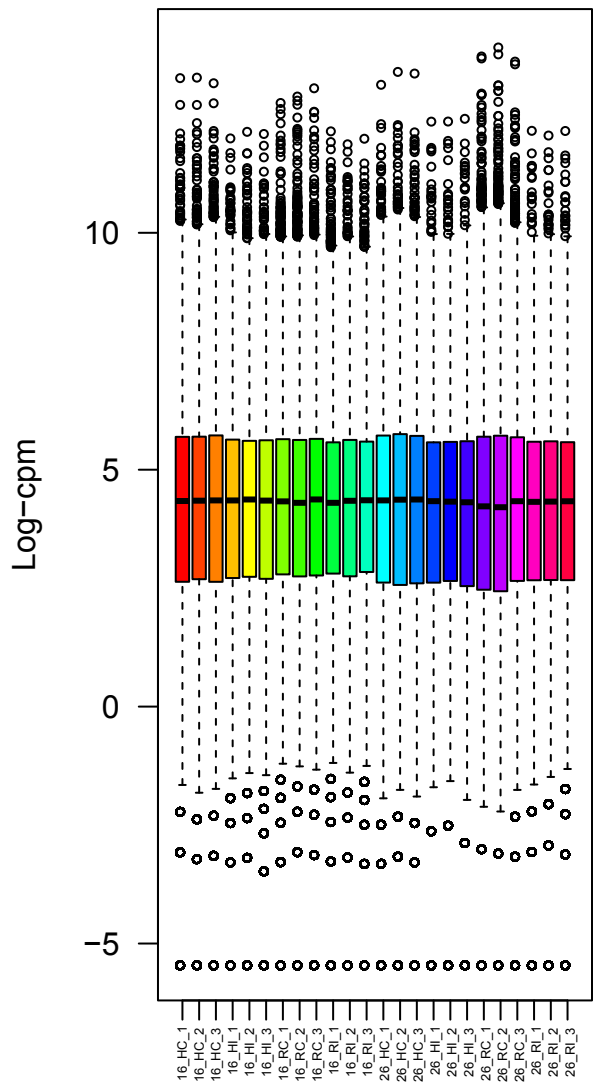

Supplement: Additional file 3: — Effect of CPM normalization on genes expression profiles. Boxplots representing the expression distribution of the expressed genes (filtered) before and after CPM normalization using TMM method for Normalization Factor calculation. After normalization, the distribution of genes expression of the 24 analyzed samples is similar. (PDF 373 kb) [file 12870_2016_941_MOESM3_ESM.pdf]

# Unsupervised clustering of sample groups

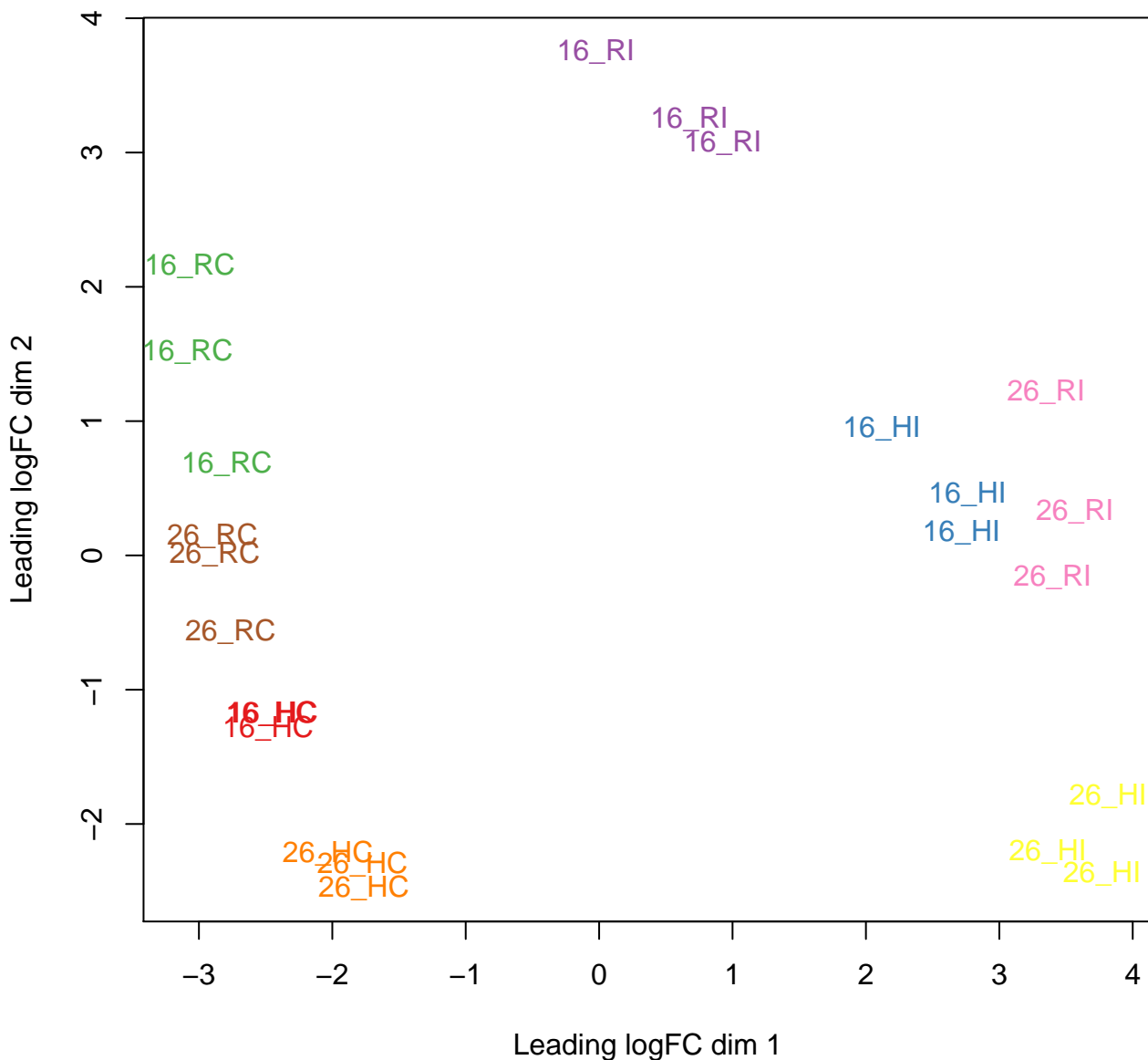

Supplement: Additional file 4: — Assessment of the biological replicates reproducibility. An unsupervised clustering of sample groups has been performed to verify the likeliness of the biological replicates for each condition. Most groups are correlated and no outlier is detected. All replicates have then been kept for differentially expressed genes analysis. (PDF 8 kb) [file 12870_2016_941_MOESM4_ESM.pdf]
